# Diammonium diaqua­bis(methyl­enediphospho­nato-*κ* ^2^ *O*,*O*′)cobaltate(II)

**DOI:** 10.1107/S1600536809042159

**Published:** 2009-10-17

**Authors:** K. A. Van der Merwe, Hendrik G. Visser, J. A. Venter

**Affiliations:** a Department of Chemistry, University of the Free State, PO Box 339, Bloemfontein, 9330, South Africa

## Abstract

In the salt, (NH_4_)_2_[Co(CH_4_O_6_P_2_)_2_(H_2_O)_2_], the methyl­ene­diphospho­nate acts as a bidentate ligand and the Co^II^ ion (site symmetry 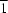) assumes an octa­hedral CoO_6_ coordination geometry. The acid H atom of the ligand is distributed over two O atoms. In the crystal, a three-dimensional network is formed through  O—H⋯O and N—H⋯O hydrogen bonds between the cations and anions.

## Related literature

For related structures, see: DeLaMatter *et al.* (1973 ▶); Jurisson *et al.* (1983 ▶); Barthelet *et al.* (2002 ▶); Stahl *et al.* (2006 ▶).
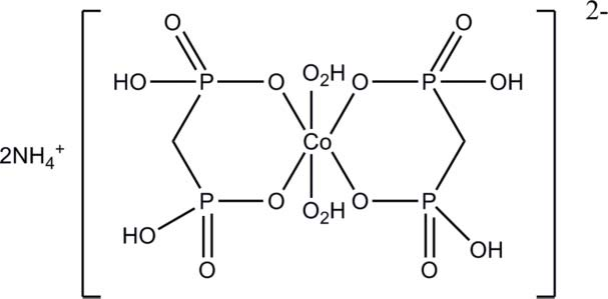


## Experimental

### 

#### Crystal data

(NH_4_)_2_[Co(CH_4_O_6_P_2_)_2_(H_2_O)_2_]
*M* *_r_* = 479.01
Triclinic, 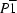

*a* = 7.455 (5) Å
*b* = 7.560 (5) Å
*c* = 8.035 (5) Å
α = 88.282 (5)°
β = 62.450 (5)°
γ = 71.834 (5)°
*V* = 378.0 (4) Å^3^
*Z* = 1
Mo *K*α radiation
μ = 1.63 mm^−1^
*T* = 103 K
0.28 × 0.16 × 0.11 mm

#### Data collection

Bruker SMART diffractometer
Absorption correction: multi-scan (*SADABS*; Sheldrick, 1996 ▶) *T*_min_ = 0.739, *T*_max_ = 0.830
8419 measured reflections
1875 independent reflections
1735 reflections with *I* > 2σ(*I*)
*R*_int_ = 0.029

#### Refinement

*R*[*F* ^2^ > 2σ(*F* ^2^)] = 0.026
*wR*(*F* ^2^) = 0.097
*S* = 1.23
1875 reflections
134 parameters
6 restraints
H atoms treated by a mixture of independent and constrained refinement
Δρ_max_ = 0.58 e Å^−3^
Δρ_min_ = −0.48 e Å^−3^

### 

Data collection: *SMART* (Bruker, 2004 ▶); cell refinement: *SAINT* (Bruker, 2004 ▶); data reduction: *SAINT*; program(s) used to solve structure: *SHELXS97* (Sheldrick, 2008 ▶); program(s) used to refine structure: *SHELXL97* (Sheldrick, 2008 ▶); molecular graphics: *DIAMOND* (Brandenberg & Putz, 2005 ▶); software used to prepare material for publication: *SHELXL97*.

## Supplementary Material

Crystal structure: contains datablocks global, I. DOI: 10.1107/S1600536809042159/ng2665sup1.cif

Structure factors: contains datablocks I. DOI: 10.1107/S1600536809042159/ng2665Isup2.hkl

Additional supplementary materials:  crystallographic information; 3D view; checkCIF report

## Figures and Tables

**Table 1:** Hydrogen-bond geometry (Å, °)

*D*—H⋯*A*	*D*—H	H⋯*A*	*D*⋯*A*	*D*—H⋯*A*
O1—H1*A*⋯O5^i^	0.819 (18)	1.99 (2)	2.795 (3)	166 (4)
O1—H1*B*⋯O4^ii^	0.835 (19)	1.994 (19)	2.827 (3)	175 (4)
N1—H1*N*⋯O7^iii^	0.825 (18)	2.09 (2)	2.892 (3)	166 (3)
N1—H2*N*⋯O6	0.825 (18)	1.98 (2)	2.796 (3)	171 (4)
N1—H3*N*⋯O3^iv^	0.821 (18)	2.17 (2)	2.959 (3)	161 (3)
N1—H4*N*⋯O3^v^	0.821 (18)	2.146 (19)	2.966 (3)	177 (3)
O6—H6*A*⋯O6^vi^	0.82	1.66	2.463 (4)	165
O7—H7⋯O4^vii^	0.82	1.66	2.435 (3)	156

Symmetry codes: (i) 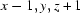; (ii) 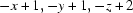; (iii) 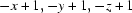; (iv) 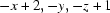; (v) 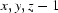; (vi) 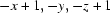; (vii) 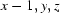.

**Fig. 1.**
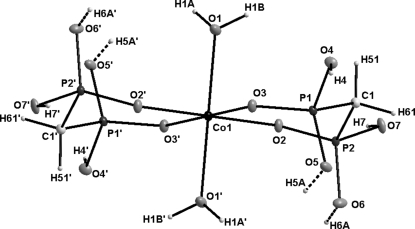
View of (I) (50% probability displacement ellipsoids). The ammonium cations have been omitted for clarity.

## supplementary crystallographic information

### Comment

Diphosphonic acids are useful for the synthesis of metalorganic frameworks exhibiting microporous properties (Barthelet *et al.*, 2002).

The Co^II^ ion in the title complex, (NH_4_)_2_[Co(C_2_H_8_O_12_)_2_(H_2_O)_2_], is in a slightly distorted octahedral environment with O—Co—O bonding angles ranging from 84.72 (8) to 95.28 (8) *^o^*. All the bonding distances and angles fall within the normal range observed for complexes of this nature (DeLaMatter *et al.*, 1973; Jurisson *et al.*, 1983 and Stahl *et al.*, 2006). In the bidentate ligand, the distances of uncoordinated O atoms to their respective P atoms do not vary much with values ranging from 1.5304 (19) and 1.5469 (19) Å. This, together with the fact that the best fits of our data were obtained when a 50% positional disorder was applied to the hydrogen atoms bonded to these O atoms, probably is enough to validate the slight disorder. A three-dimensional network is provided by numerous hydrogen bonds between the oxygen atoms of the anionic species and the ammonium cations.

### Experimental

CoCl_2_.6H_2_O (0,1696 g, 0,00071 mol) was dissolved in water (7 cm^3^) and heated to 70°C. The pH of the solution was 4,89 and deep pink in colour. Ammonium bicarbonate was added to raise the pH to 5,61 after which methylene disphosphonate (0,25 g, 0,00142 mol), dissolved in water (5 cm^3^) was added dropwise. The final pH of the solution was adjusted to 1.50 with ammonium bicarbonate. (Yield: 84.1%)

### Refinement

The aliphatic H atoms were placed in geometrically idealized positions and constrained to ride on its parent atoms with U<i/>_iso_(H) = 1.2U<i/>_eq_(C).   The hydroxyl H atoms were placed in geometrically idealized positions and constrained to ride on its parent atoms with U<i/>_iso_(H) = 1.5U<i/>_eq_(O).  A 50% positional disorder was assigned to these hydrogen atoms and provided the best fits of the data. The highest electron density lies within 0.74 Å from O7.

### Crystal data

**Table:**

(NH_4_)_2_[Co(CH_4_O_6_P_2_)_2_(H_2_O)_2_]	*Z* = 1
*M**_r_* = 479.01	*F*(000) = 237
Triclinic, *P*1	*D*_x_ = 2.069 Mg m^−^^3^
Hall symbol: -P 1	Mo *K*α radiation, λ = 0.71069 Å
*a* = 7.455 (5) Å	Cell parameters from 4649 reflections
*b* = 7.560 (5) Å	θ = 3.2–28.3°
*c* = 8.035 (5) Å	µ = 1.63 mm^−^^1^
α = 88.282 (5)°	*T* = 103 K
β = 62.450 (5)°	Rod, pink
γ = 71.834 (5)°	0.28 × 0.16 × 0.11 mm
*V* = 378.0 (4) Å^3^	

### Data collection

**Table:**

Bruker SMART diffractometer	1875 independent reflections
Radiation source: sealed tube	1735 reflections with *I* > 2σ(*I*)
graphite	*R*_int_ = 0.029
φ and ω scans	θ_max_ = 28.4°, θ_min_ = 3.2°
Absorption correction: multi-scan (*SADABS*; Sheldrick, 1996)	*h* = −9→9
*T*_min_ = 0.739, *T*_max_ = 0.830	*k* = −9→10
8419 measured reflections	*l* = −10→9

### Refinement

**Table:**

Refinement on *F*^2^	6 restraints
Least-squares matrix: full	H atoms treated by a mixture of independent and constrained refinement
*R*[*F*^2^ > 2σ(*F*^2^)] = 0.026	*w* = 1/[σ^2^(*F*_o_^2^) + (0.0501*P*)^2^ + 0.4031*P*] where *P* = (*F*_o_^2^ + 2*F*_c_^2^)/3
*wR*(*F*^2^) = 0.097	(Δ/σ)_max_ < 0.001
*S* = 1.23	Δρ_max_ = 0.58 e Å^−^^3^
1875 reflections	Δρ_min_ = −0.48 e Å^−^^3^
134 parameters	

### Special details

**Table:**

Geometry. All e.s.d.'s (except the e.s.d. in the dihedral angle between two l.s. planes) are estimated using the full covariance matrix. The cell e.s.d.'s are taken into account individually in the estimation of e.s.d.'s in distances, angles and torsion angles; correlations between e.s.d.'s in cell parameters are only used when they are defined by crystal symmetry. An approximate (isotropic) treatment of cell e.s.d.'s is used for estimating e.s.d.'s involving l.s. planes.

### Fractional atomic coordinates and isotropic or equivalent isotropic displacement parameters (Å^2^)

**Table:**

	*x*	*y*	*z*	*U*_iso_*/*U*_eq_	Occ. (<1)
Co1	0.5	0	1	0.00601 (14)	
P1	0.79673 (9)	0.26243 (8)	0.74506 (8)	0.00547 (16)	
P2	0.40236 (9)	0.26654 (8)	0.71287 (8)	0.00548 (15)	
C1	0.5577 (4)	0.3956 (3)	0.7338 (3)	0.0069 (4)	
H51	0.4658	0.487	0.847	0.008*	
H61	0.5984	0.465	0.6264	0.008*	
O2	0.3230 (3)	0.1616 (2)	0.8790 (2)	0.0082 (3)	
O3	0.7476 (3)	0.1227 (2)	0.8847 (2)	0.0079 (3)	
O6	0.5476 (3)	0.1357 (2)	0.5238 (2)	0.0103 (4)	
H6A	0.4935	0.058	0.5191	0.016*	0.5
O1	0.3253 (3)	0.2144 (3)	1.2321 (2)	0.0101 (4)	
N1	0.8609 (4)	0.1904 (3)	0.1800 (3)	0.0114 (4)	
O7	0.2170 (3)	0.4231 (2)	0.7030 (2)	0.0092 (3)	
H7	0.1063	0.3971	0.7569	0.014*	0.5
O5	0.9736 (3)	0.1640 (2)	0.5452 (2)	0.0091 (3)	
H5A	0.9708	0.0583	0.5301	0.014*	0.5
O4	0.8575 (3)	0.4110 (2)	0.8125 (2)	0.0083 (3)	
H4	0.9866	0.3715	0.7787	0.013*	0.5
H1N	0.860 (5)	0.298 (3)	0.197 (4)	0.010 (8)*	
H2N	0.779 (5)	0.165 (6)	0.282 (4)	0.029 (10)*	
H3N	0.984 (3)	0.121 (4)	0.149 (5)	0.020 (9)*	
H4N	0.829 (6)	0.175 (5)	0.098 (4)	0.020 (9)*	
H1A	0.233 (5)	0.201 (5)	1.334 (3)	0.020 (9)*	
H1B	0.274 (6)	0.323 (3)	1.212 (5)	0.027 (10)*	

### Atomic displacement parameters (Å^2^)

**Table:**

	*U*^11^	*U*^22^	*U*^33^	*U*^12^	*U*^13^	*U*^23^
Co1	0.0060 (2)	0.0058 (2)	0.0061 (2)	−0.00256 (17)	−0.00242 (18)	0.00160 (16)
P1	0.0050 (3)	0.0051 (3)	0.0066 (3)	−0.0025 (2)	−0.0026 (2)	0.0016 (2)
P2	0.0056 (3)	0.0052 (3)	0.0062 (3)	−0.0025 (2)	−0.0028 (2)	0.0017 (2)
C1	0.0074 (11)	0.0060 (10)	0.0083 (10)	−0.0026 (8)	−0.0043 (9)	0.0013 (8)
O2	0.0077 (8)	0.0087 (8)	0.0093 (8)	−0.0042 (6)	−0.0043 (7)	0.0044 (6)
O3	0.0071 (8)	0.0083 (8)	0.0087 (8)	−0.0038 (6)	−0.0036 (6)	0.0034 (6)
O6	0.0115 (9)	0.0112 (8)	0.0081 (8)	−0.0071 (7)	−0.0023 (7)	−0.0008 (6)
O1	0.0107 (9)	0.0075 (8)	0.0085 (8)	−0.0032 (7)	−0.0017 (7)	0.0011 (6)
N1	0.0107 (11)	0.0102 (10)	0.0108 (10)	−0.0006 (8)	−0.0051 (9)	0.0010 (8)
O7	0.0077 (8)	0.0076 (8)	0.0147 (9)	−0.0033 (6)	−0.0069 (7)	0.0055 (6)
O5	0.0086 (8)	0.0067 (8)	0.0083 (8)	−0.0033 (6)	−0.0006 (6)	−0.0003 (6)
O4	0.0056 (8)	0.0087 (8)	0.0113 (8)	−0.0026 (6)	−0.0042 (7)	0.0002 (6)

### Geometric parameters (Å, °)

**Table:**

Co1—O2	2.0714 (18)	P2—C1	1.793 (2)
Co1—O2^i^	2.0714 (18)	C1—H51	0.97
Co1—O1^i^	2.102 (2)	C1—H61	0.97
Co1—O1	2.102 (2)	O6—H6A	0.82
Co1—O3^i^	2.135 (2)	O1—H1A	0.819 (18)
Co1—O3	2.135 (2)	O1—H1B	0.835 (19)
P1—O3	1.5166 (18)	N1—H1N	0.825 (18)
P1—O4	1.5304 (19)	N1—H2N	0.825 (18)
P1—O5	1.5403 (19)	N1—H3N	0.821 (18)
P1—C1	1.795 (3)	N1—H4N	0.821 (18)
P2—O2	1.5032 (18)	O7—H7	0.82
P2—O6	1.5363 (19)	O5—H5A	0.82
P2—O7	1.5469 (19)	O4—H4	0.82
			
O2—Co1—O2^i^	180	O2—P2—C1	111.74 (11)
O2—Co1—O1^i^	92.53 (8)	O6—P2—C1	106.30 (11)
O2^i^—Co1—O1^i^	87.47 (8)	O7—P2—C1	102.44 (12)
O2—Co1—O1	87.47 (8)	P2—C1—P1	116.96 (14)
O2^i^—Co1—O1	92.53 (8)	P2—C1—H51	108.1
O1^i^—Co1—O1	180.0000 (10)	P1—C1—H51	108.1
O2—Co1—O3^i^	84.72 (8)	P2—C1—H61	108.1
O2^i^—Co1—O3^i^	95.28 (8)	P1—C1—H61	108.1
O1^i^—Co1—O3^i^	88.91 (8)	H51—C1—H61	107.3
O1—Co1—O3^i^	91.09 (8)	P2—O2—Co1	126.71 (11)
O2—Co1—O3	95.28 (8)	P1—O3—Co1	133.61 (10)
O2^i^—Co1—O3	84.72 (8)	P2—O6—H6A	109.5
O1^i^—Co1—O3	91.09 (8)	Co1—O1—H1A	121 (2)
O1—Co1—O3	88.91 (8)	Co1—O1—H1B	117 (3)
O3^i^—Co1—O3	180.00 (7)	H1A—O1—H1B	106 (4)
O3—P1—O4	111.68 (11)	H1N—N1—H2N	108 (4)
O3—P1—O5	111.77 (11)	H1N—N1—H3N	105 (3)
O4—P1—O5	110.47 (11)	H2N—N1—H3N	108 (4)
O3—P1—C1	109.61 (11)	H1N—N1—H4N	114 (3)
O4—P1—C1	103.92 (11)	H2N—N1—H4N	111 (4)
O5—P1—C1	109.08 (11)	H3N—N1—H4N	110 (3)
O2—P2—O6	112.62 (11)	P2—O7—H7	109.5
O2—P2—O7	112.39 (10)	P1—O5—H5A	109.5
O6—P2—O7	110.73 (11)	P1—O4—H4	109.5

Symmetry codes: (i) −*x*+1, −*y*, −*z*+2.

### Hydrogen-bond geometry (Å, °)

**Table:**

*D*—H···*A*	*D*—H	H···*A*	*D*···*A*	*D*—H···*A*
O1—H1A···O5^ii^	0.82 (2)	1.99 (2)	2.795 (3)	166 (4)
O1—H1B···O4^iii^	0.84 (2)	1.99 (2)	2.827 (3)	175 (4)
N1—H1N···O7^iv^	0.83 (2)	2.09 (2)	2.892 (3)	166 (3)
N1—H2N···O6	0.83 (2)	1.98 (2)	2.796 (3)	171 (4)
N1—H3N···O3^v^	0.82 (2)	2.17 (2)	2.959 (3)	161 (3)
N1—H4N···O3^vi^	0.82 (2)	2.15 (2)	2.966 (3)	177 (3)
O6—H6A···O6^vii^	0.82	1.66	2.463 (4)	165
O7—H7···O4^viii^	0.82	1.66	2.435 (3)	156

Symmetry codes: (ii) *x*−1, *y*, *z*+1; (iii) −*x*+1, −*y*+1, −*z*+2; (iv) −*x*+1, −*y*+1, −*z*+1; (v) −*x*+2, −*y*, −*z*+1; (vi) *x*, *y*, *z*−1; (vii) −*x*+1, −*y*, −*z*+1; (viii) *x*−1, *y*, *z*.
